# A two step Bayesian approach for genomic prediction of breeding values

**DOI:** 10.1186/1753-6561-6-S2-S12

**Published:** 2012-05-21

**Authors:** Mohammad M Shariati, Peter Sørensen, Luc Janss

**Affiliations:** 1 Department of Molecular Biology and Genetics, Faculty of Science and Technology, Aarhus University, DK-8830 Tjele, Denmark; 2 Department of Animal Science, Faculty of Agriculture, Ferdowsi University of Mashhad, 91775 Mashhad, Iran

## Abstract

**Background:**

In genomic models that assign an individual variance to each marker, the contribution of one marker to the posterior distribution of the marker variance is only one degree of freedom (df), which introduces many variance parameters with only little information per variance parameter. A better alternative could be to form clusters of markers with similar effects where markers in a cluster have a common variance. Therefore, the influence of each marker group of size *p *on the posterior distribution of the marker variances will be *p *df.

**Methods:**

The simulated data from the 15^th ^QTL-MAS workshop were analyzed such that SNP markers were ranked based on their effects and markers with similar estimated effects were grouped together. In step 1, all markers with minor allele frequency more than 0.01 were included in a SNP-BLUP prediction model. In step 2, markers were ranked based on their estimated variance on the trait in step 1 and each 150 markers were assigned to one group with a common variance. In further analyses, subsets of 1500 and 450 markers with largest effects in step 2 were kept in the prediction model.

**Results:**

Grouping markers outperformed SNP-BLUP model in terms of accuracy of predicted breeding values. However, the accuracies of predicted breeding values were lower than Bayesian methods with marker specific variances.

**Conclusions:**

Grouping markers is less flexible than allowing each marker to have a specific marker variance but, by grouping, the power to estimate marker variances increases. A prior knowledge of the genetic architecture of the trait is necessary for clustering markers and appropriate prior parameterization.

## Background

The statistical methods for genomic selection introduced by Meuwissen et al [1]; i.e. SNP-BLUP, BayesA and BayesB, are still the most popular ones. G-BLUP that exploits SNP genotypes to build genomic relationship matrix emerged some years later and it was shown that G-BLUP and SNP-BLUP are equivalent models [2,3]. From other approaches for genomic prediction of breeding values (BV) one can name LASSO [4], Bayesian LASSO [5] and reproducing Hilbert space models [6]. These latter methods have not led to magnificent improvement over the original methods proposed by Meuwissen et al. [1] in BV prediction.

In BayesA and BayesB the prior distribution of each marker effect is assumed normal with a marker specific variance $\sigma_{i}^{2}$. The marker variances follow a scaled-inverse Chi-squared distribution with some scale and degrees of freedom (df) hyperparameters, a priori [1]. Therefore, the marginal posterior distribution of marker effects is a t-distribution [7]. In this setting, the resulted drawback is that the contribution of each marker to the posterior distribution of marker variances is only one df, that is very little information for any prior specification [7]. As a remedy, Gianola et al [7] suggested to form clusters of markers where markers in a cluster have a common variance. Therefore, the influence of each marker group of size *p *on the posterior distribution of the marker variances will be *p *df. Further, they suggested assigning noninformative priors to scale and df parameters of the distribution of marker variance or marker-group variance. In the present study, a strategy for grouping markers with similar effects together was applied to the simulated data from the 15th QTL-MAS workshop [8]. The analysis was performed in two steps, where, in the first step marker effects were estimated in a SNP-BLUP analysis, and in the next step, markers with similar effects were allocated to one group. From complexity point of view, this strategy stands between SNP-BLUP where a single variance is common to all markers, and BayesA-B, where each marker has a variance. The aim of this study was to investigate the performance of this strategy on the accuracy of genomic breeding values. Further, the effect of prior setting for the marker-group variances on the extent of phenotypic variance explained by each group of markers was investigated.

## Methods

### Model

First, an animal model BLUP using pedigree and phenotypes was performed to predict breeding values of all animals, and REML estimate of heritability was obtained [9]. Further, the heritability and breeding values were estimated using genomic models [10] and compared with the ones from animal model.

The SNP-BLUP additive genomic model was used to estimate SNP effects in the first step as:

(1) $$y_{i}=\mu +\underset{j}{\sum }x_{ij}\beta_{j}+e_{i}\;i=\text{ 1},\ldots ,n\;\text{and}\;j=\text{ 1},\ldots ,q$$

where *μ *is the general mean,* β_j _*is the allele substitution effect of the *j*th SNP with $\beta \sim N\left(0,I\sigma_{\beta }^{2}\right)$, *x_ij _*is the genotype covariate of the *j*th marker for the *i*th animal, associating marker effects *β_j _*to the phenotype *y_i_*, and ***e**_i _*is residual or environmental effect with $e\sim N\left(0,I\sigma_{e}^{2}\right)$. The genotype covariate is initially coded as 0,1,2 for homozygote, heterozygote and alternate homozygote, and then centered to have mean zero. Flat priors were considered for the residual variance $\sigma_{e}^{2}$ and marker variance $\sigma_{\beta }^{2}$ to estimate them in a reference Bayesian approach that uses the frequentist likelihood as the Bayesian posterior distribution.

The estimated marker effect ${\hat{\beta }}_{j}$ from model (1) was used to estimate the variance explained by that marker in the population as $p_{j}\left(\text{1}-p_{j}\right)\beta_{j}^{2}$, where *p_j _*is the frequency of one of the alleles of the *j*th SNP. Then, all markers were sorted based on their explained variance and each 150 marker were grouped together. We tried a grid of different SNP-group sizes and among them a SNP-group size of 150 yielded the highest accuracy of PBV in the validation dataset (explained in *results and discussion*).The model for the second step (ALL-SNP) with grouped SNP and group specific SNP variance was:

(2) $$y_{i}=\mu +\underset{j}{\sum }x_{ij}\beta_{jk}+e_{i}\;i=\text{ 1},\ldots ,n,\;j=\text{1},\ldots ,q\;\text{and}\;k=\text{1},\ldots ,g$$

where *β_jk _*denotes the effect of the *j*th marker that belong to group *k*, and *g *is the total number of groups. A model was used with a variance parameter per group, and the group variances are jointly modeled to have an inverse chi-square distribution in which the scale is treated as a model parameter. The prior specification was as follows:

*μ~uniform*; $\beta_{jk}\sim N\left(0,\sigma_{k}^{2}\right)$; $\sigma_{k}^{2}\sim \chi^{-2}\left(scale,df\right)$; $\sigma_{e}^{2}\sim uniform\left(>0\right)$; $\sigma_{e}^{2}\sim uniform\left(>0\right)$; *scale~uniform*(>0); and *df*: a fixed number as hyperparameter.

The fully conditional posterior distributions were as follows:

$$\begin{array}{c} \mu |.\sim N\left(n^{-1}\overset{n}{\underset{i=1}{\sum }}\left(y_{i}-\overset{q}{\underset{j=1}{\sum }}x_{ij}\beta_{jk}\right),\frac{\sigma_{e}^{2}}{n}\right);\\ \beta_{jk}|.\sim N\left({\left[\overset{n}{\underset{i=1}{\sum }}x_{ij}^{2}+\frac{\sigma_{e}^{2}}{\sigma_{k}^{2}}\right]}^{-1}\overset{n}{\underset{i=1}{\sum }}x_{ij}^{}\left(y_{i}-\mu -\overset{q}{\underset{l\neq j}{\sum }}x_{il}\beta_{lk}\right),{\left[\overset{n}{\underset{i=1}{\sum }}x_{ij}^{2}+\frac{\sigma_{e}^{2}}{\sigma_{k}^{2}}\right]}^{-1}\sigma_{e}^{2}\right); \end{array}$$

$\sigma_{k}^{2}|.\sim \chi^{-2}\left({\left[n_{k}+df\right]}^{-1}\left[n_{k}\overset{n_{k}}{\underset{j=1}{\sum }}\beta_{jk}^{2}+scale\times df\right],n_{k}+df\right)$ where *n_k _*is the group size for SNP-group *k*. In this study *n_k _*was equal to *p *for all SNP-groups;

(3) $$\begin{array}{c} \sigma_{e}^{2}|.\sim \chi^{-2}\left({\left[n-2\right]}^{-1}\left[n\left(\overset{n}{\underset{i=1}{\sum }}y_{i}-\mu -\overset{q}{\underset{j=1}{\sum }}x_{ij}\beta_{jk}\right)\right],n-2\right);\\ \text{and}\;scale|.\sim Gamma\left(\frac{g\times df}{2}+1,\frac{2}{df}\overset{g}{\underset{k=1}{\sum }}\frac{1}{\sigma_{k}^{2}}\right). \end{array}$$

In further analyses, respectively, 1500 (SNP1500) and 450 (SNP450a, SNP450b) markers with the largest effects from model (2) were selected and were allocated to groups of size 150 (for 1500 markers), and 75 or 50 (for 450 markers). Then, breeding values of animals without records were predicted using the marker effects from these subsets of markers.

### Gibbs sampling

Gibbs sampling was used to sample from joint posterior distributions for all datasets. The chain length was 50.000 in all analyses where the first 20.000 samples were discarded as burn in and one of each 30 samples were saved to compute the posterior means for the parameters. Preliminary experience showed that a burn in of size 20.000 guarantees the convergence for different parameters.

## Results and discussion

The challenge was to predict the breeding values of 1000 genotyped animals with no phenotypes. The available data comprised of 2000 animals with both genotype and phenotype. We validated the models using 200 animals such that the last progeny with genotype and phenotype record from each dam was taken out of the data and used for validation. The remaining 1800 animals were used to train the model. Based on this validation the size of the SNP-groups was chosen to be 150 and the scale and df of the prior distribution of marker variances were set to zero because this resulted in the highest accuracy for the validation animals. A scale and df setting of zero corresponds to the so-called Jeffreys or non-informative prior for variances. After the true breeding values of the other 1000 animals were provided, it turned out that other prior specification for the marker variances can give better predictive abilities. Perhaps, the reason was that the 200 validation animals were not enough to represent the whole population. Further, there was an imprinted QTL where the effect is expressed if it has been transmitted from one of the parents only. It is likely that among these 200 animals most of them or all of them have got the paternal (maternal) imprinted QTL. Further details of the impact of prior specification of the marker variances on estimation of SNP effects and breeding values will be discussed.

### Accuracy of predicted breeding values

The accuracies of predicted breeding values (PBV) from the two-step method were higher than PBV from animal model BLUP and SNP-BLUP (Table 1). Among the two-step strategies, including all SNP in the model yielded highest accuracy followed by, respectively, including 1500 and 450 markers. Animal model BLUP yielded the lowest accuracy of PBV because it takes only parent average BV to predict BV of offspring. Clustering SNP in groups with similar effects improved the accuracy compared to SNP-BLUP by around 4%, but the accuracy was still 7% lower than BayesB method [11,12]. Given that only eight makers were causative QTL, it is natural that BayesB performs best because it has been invented to locate the marker variance efficiently for few QTL with large effects [1]. In the two-step approaches, all of causative SNP were allocated to the first group with largest effect but the group size was much larger than the true number of causative SNP. This can lead to some discrepancy in estimating SNP effects that is described later in this paper.

**Table 1:** Correlation between predicted breeding values of unphenotyped animals and their true genetic values or expected genetic values of their progeny

Method	Genetic value	Progeny value
BLUP	0.608	0.595
SNP-BLUP	0.825	0.822
All_SNP^1^	0.862	0.841
SNP1500^1^	0.861	0.840
SNP450a^2^	0.856	0.830
SNP450b^3^	0.854	0.823

^1, 2, 3 ^Group sizes were 150, 75 and 50, respectively.

### Variance components and heritability

Table 2 shows the estimates of heritability of the trait using animal model BLUP (REML), SNP-BLUP and grouping scenarios. The REML estimate of (narrow sense) heritability was very close to the simulated (broad sense) heritability. This indicates that the non-additive variance due to one pair of epistatic QTL has been negligible. SNP-BLUP underestimated heritability by 1% meaning that it has captured most of additive variance despite the fact that genetic values were due to few QTL with large effects. Several studies have shown that SNP-BLUP can capture relationship; i,e, genetic similarities between animals, and this is independent of the number of QTL and the distribution of QTL effects affecting the trait [13,14]. This characteristic of SNP-BLUP has led to the use of genomic relationship matrix in genetic evaluation programs based on SNP markers [2]. On the other hand, all two-step scenarios overestimated heritability by around 5.5%. This shows that they were not able to separate the signal from the noise perfectly.

**Table 2:** Estimates of heritability from different methods

Method	Heritability
True	0.300
REML	0.297
SNP-BLUP	0.289
All_SNP^1^	0.355
SNP1500^1^	0.355
SNP450a^2^	0.357
SNP450b^3^	0.356

^1, 2, 3 ^Group sizes were 150, 75 and 50, respectively.

### Prior distribution for variances

**Figure 1:**
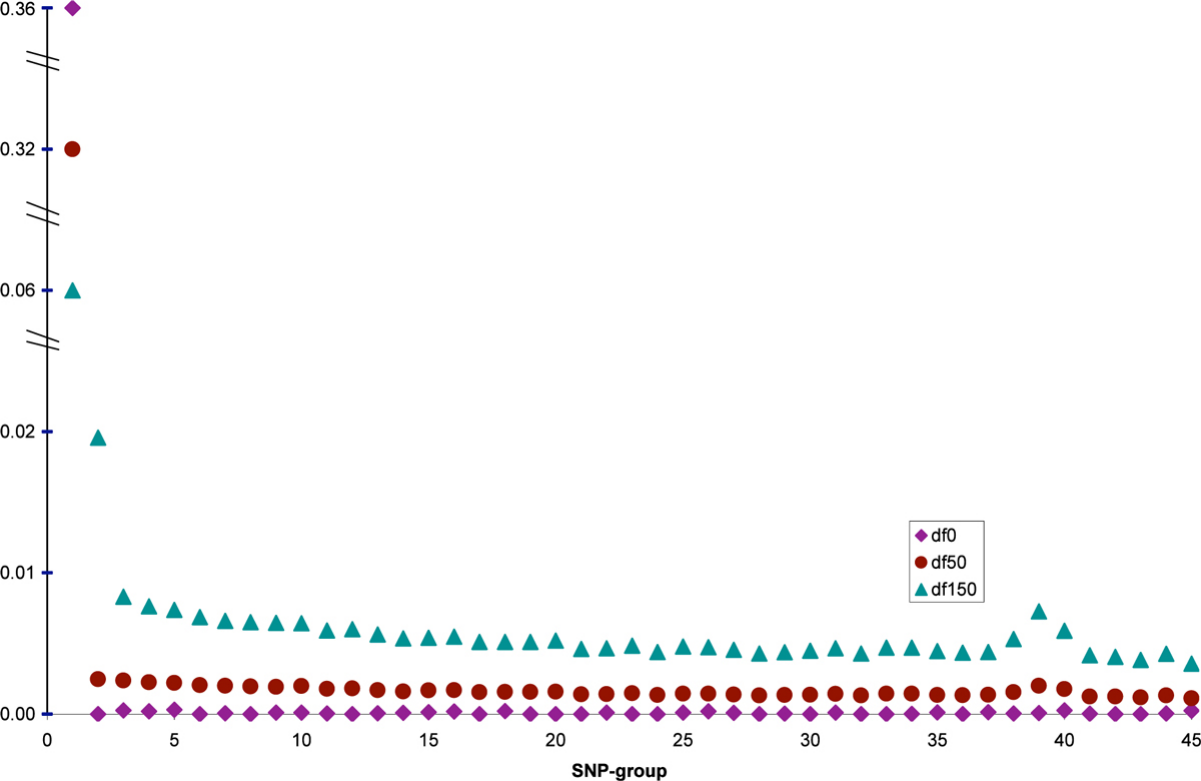
**Marker-group heritabilities for different prior degrees of freedom for the group variances**. Grouping was such that the first group was consisted of markers with largest effects in the SNP-BLUP analysis and similarly the last group was consisted of markers with the smallest effects on the trait.

Overestimation of the heritability in the two-step method was mainly due to the prior setting for the SNP-group variances (scale = 0, df = 0, corresponding to the Jeffreys or non-informative prior). In order to investigate the effect of prior df, two other analyses with either 50 or 150 degrees of freedom were run with all markers (extensions of ALL-SNP), where, the scale parameter was updated using equation (3). Figure 1 shows the SNP-group heritabilities for different df for the prior distribution of marker variances. The overall heritabilities were 0.355, 0.351 and 0.305, respectively, for the df of 0, 50 and 150. When both scale and df were set to zero, which is the non-informative prior distribution ${\mathrm{(\sigma}}_{k}^{2}\sim 1/\sigma_{k}^{2}$), the first SNP-group with largest QTL had a huge variance that explained all the genetic variation plus a large fraction of noise. This setting resembles a fixed regression scenario were in the first group, the large variance induces very little shrinkage for 150 SNP in this group. Because the SNP in the first group were not all the real QTL, the model overestimated the genetic variance. For df of 150, a priori, the posterior df was equal to 300 and the impact of 150 SNP within a group in determining the group variance reduced to half and the other half was the share of prior. Therefore, a harder shrinkage on the first group and less shrinkage on the rest of groups was performed. It can be said that there is a trade off between the accuracy of PBV and unbiased estimation of heritability. A model with high df for the prior distribution of group variances performs similar to a SNP-BLUP model; it yields unbiased estimate of the heritability but due to strong shrinkage on all markers, the accuracy of PBV will not be high. On the other hand, a very small df can lead to a regression with fixed marker effects that is prone to capture noise.

## Conclusions

Grouping markers is less flexible than allowing each marker to have a specific marker variance but, by grouping, the power to estimate marker variances increases. A prior knowledge of the genetic architecture of the trait is necessary in order to clustering markers and appropriate prior parameterization. In the workshop data set, the presented approach to group SNPs gave better predictions than a SNP-BLUP model, but worse predictions than a mixture (BayesB type) model. However, the workshop data set had a limited amount of QTL, which may not be representative for many real data sets. In real data often little advantages are seen for mixture models compared to SNP-BLUP, and as our method clearly outperformed SNP-BLUP our method could be of interest for further study in real data.

## Competing interests

The authors declare that they have no competing interests.

## Authors' contributions

MMS analyzed the data and drafted the paper. All authors contributed in planning the study, discussing the results and reading and editing the paper.
